# In search of convergent regional brain abnormality in cognitive emotion regulation: A transdiagnostic neuroimaging meta‐analysis

**DOI:** 10.1002/hbm.25722

**Published:** 2021-11-26

**Authors:** Tina Khodadadifar, Zahra Soltaninejad, Amir Ebneabbasi, Claudia R. Eickhoff, Christian Sorg, Thilo Van Eimeren, Kai Vogeley, Mojtaba Zarei, Simon B. Eickhoff, Masoud Tahmasian

**Affiliations:** ^1^ School of Cognitive Sciences Institute for Research in Fundamental Sciences Tehran Iran; ^2^ Institute of Medical Science and Technology Shahid Beheshti University Tehran Iran; ^3^ Cognitive and Brain Science Institute Shahid Beheshti University Tehran Iran; ^4^ Institute of Neuroscience and Medicine, Brain and Behaviour (INM‐7) Research Center Jülich Jülich Germany; ^5^ Institute of Clinical Neuroscience and Medical Psychology Heinrich Heine University Düsseldorf Düsseldorf Germany; ^6^ Institute of Neuroscience and Medicine, Structural and functional organization of the brain (INM‐1) Research Center Jülich Jülich Germany; ^7^ TUM‐Neuroimaging Center (TUM‐NIC), Klinikum Rechts der Isar Technische Universität München Munich Germany; ^8^ Department of Neuroradiology Klinikum Rechts der Isar, Technische Universität München Munich Germany; ^9^ Department of Psychiatry and Psychotherapy Klinikum Rechts der Isar, Technische Universität München Munich Germany; ^10^ Multimodal Neuroimaging Group, Department of Nuclear Medicine, Faculty of Medicine and University Hospital of Cologne University of Cologne Cologne Germany; ^11^ Department of Neurology, Faculty of Medicine and University Hospital of Cologne University of Cologne Cologne Germany; ^12^ Department of Psychiatry and Psychotherapy University Hospital Cologne Cologne Germany; ^13^ Cognitive Neuroscience (INM‐3) Institute of Neuroscience and Medicine, Research Center Jülich Jülich Germany; ^14^ Institute for Systems Neuroscience, Medical Faculty Heinrich‐Heine University Düsseldorf Düsseldorf Germany

**Keywords:** activation likelihood estimation, coordinate‐based meta‐analysis, emotion regulation, reappraisal

## Abstract

Ineffective use of adaptive cognitive strategies (e.g., reappraisal) to regulate emotional states is often reported in a wide variety of psychiatric disorders, suggesting a common characteristic across different diagnostic categories. However, the extent of shared neurobiological impairments is incompletely understood. This study, therefore, aimed to identify the transdiagnostic neural signature of disturbed reappraisal using the coordinate‐based meta‐analysis (CBMA) approach. Following the best‐practice guidelines for conducting neuroimaging meta‐analyses, we systematically searched PubMed, ScienceDirect, and Web of Science databases and tracked the references. Out of 1,608 identified publications, 32 whole‐brain neuroimaging studies were retrieved that compared brain activation in patients with psychiatric disorders and healthy controls during a reappraisal task. Then, the reported peak coordinates of group comparisons were extracted and several activation likelihood estimation (ALE) analyses were performed at three hierarchical levels to identify the potential spatial convergence: the global level (i.e., the pooled analysis and the analyses of increased/decreased activations), the experimental‐contrast level (i.e., the analyses of grouped data based on the regulation goal, stimulus valence, and instruction rule) and the disorder‐group level (i.e., the analyses across the experimental‐contrast level focused on increasing homogeneity of disorders). Surprisingly, none of our analyses provided significant convergent findings. This CBMA indicates a lack of transdiagnostic convergent regional abnormality related to reappraisal task, probably due to the complex nature of cognitive emotion regulation, heterogeneity of clinical populations, and/or experimental and statistical flexibility of individual studies.

## Graphical abstract

The present study aimed to identify the transdiagnostic neural signature of disturbed reappraisal using the coordinate‐based meta‐analysis. Our findings indicate a lack of transdiagnostic convergent regional abnormality related to reappraisal task, probably due to the complex nature of cognitive emotion regulation, heterogeneity of clinical populations, and/or experimental and statistical flexibility of individual studies.

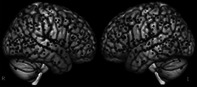

## 1 INTRODUCTION

Throughout our daily lives, we are constantly exposed to a wide range of emotionally arousing situations. Lacking the capacity to effectively use emotion regulation strategies to modify the occurrence, intensity, and duration of an emotional experience is referred to as emotion dysregulation, which can negatively affect our personal and social functioning and may cause serious mental health issues (Beauchaine & Cicchetti, 2019). Regulatory strategies are putatively considered “adaptive” or “maladaptive” based on their positive or negative associations with psychopathological symptoms (Aldao, Nolen‐Hoeksema, & Schweizer, 2010). Accordingly, healthy emotion regulation involves a balanced interplay between the use of adaptive and maladaptive strategies to reach a desired emotional state, whereas emotion dysregulation reflects unsuccessful handling of emotions caused by over‐reliance on maladaptive and/or failure in recruiting adaptive strategies (Aldao et al., 2010). It is estimated that emotion dysregulation occurs in about 40–70% of individuals diagnosed with psychiatric disorders (Jazaieri, Urry, & Gross, 2013), suggesting a transdiagnostic phenomenon (Aldao, 2016; Fernandez, Jazaieri, & Gross, 2016).

Emotion dysregulation has been extensively studied in the context of cognitive emotion regulation with a particular focus on reappraisal, an antecedent strategy that incorporates cognitive processes to alter the meaning or relevance of stimuli in order to change their emotional impact. (Aldao et al., 2010; Cludius, Mennin, & Ehring, 2020; D'Agostino, Covanti, Monti, & Starcevic, 2017; Werner & Gross, 2010). Reappraisal is a universal ability that can be used to maintain, decrease or increase negative and positive emotions (Nezlek & Kuppens, 2008). But, it is mainly required for reframing an emotionally aversive situation by creating a neutral or a more pleasant interpretation (Gross, 1998). Reappraisal has attracted attention as one of the most effective and adaptive strategies due to its immediate positive effects on emotional experience, as well as its long‐term beneficial outcomes for mental health (Hu et al., 2014; Webb, Miles, & Sheeran, 2012). Despite the health‐protective benefits of reappraisal, patients with mental illnesses generally report infrequent deployment of this regulation strategy, particularly in distressing or unpleasant situations (Aldao et al., 2010; Cludius et al., 2020; D'Agostino et al., 2017).

Lower reappraisal tendency in psychiatric patients might be related to their inability to implement this strategy in an effective way (Silvers & Moreira, 2019). Reappraisal is a top‐down and effortful process that depends on intact cognitive control and executive functioning (McRae, Jacobs, Ray, John, & Gross, 2012; Schmeichel & Tang, 2015). Thus, having trouble recruiting such higher‐order processes might lead to a decreased desire for using this strategy over time, which in turn could diminish its efficient health outcomes (Ford, Karnilowicz, & Mauss, 2017). Neuroimaging studies on reappraisal in healthy (Etkin, Büchel, & Gross, 2015; Ochsner & Gross, 2005; Ochsner, Silvers, & Buhle, 2012; Öner, 2018) and clinical populations (Green & Malhi, 2006; Silvers, Buhle, & Ochsner, 2014; Taylor & Liberzon, 2007; Zilverstand, Parvaz, & Goldstein, 2017) appear to support altered mechanisms of reappraisal across different patient groups. Accordingly, inefficient reappraisal performance is thought to be associated with a transdiagnostic pattern of aberrant brain activation in frontal cognitive control regions which are necessary to modulate the activation in regions subserving emotion generation (Zilverstand et al., 2017). Supporting this hypothesis, several transdiagnostic studies have reported the common pathways of disturbances in the neural mechanisms underlying cognitive control and executive functioning (Malloy‐Diniz, Miranda, & Grassi‐Oliveira, 2017; McTeague, Goodkind, & Etkin, 2016), emotion processing system as the regulation target (McTeague et al., 2020; Schulze, Schulze, Renneberg, Schmahl, & Niedtfeld, 2019), and their network interactions (Kebets et al., 2020).

This literature, therefore, could be construed to claim that there may exist a consistent pattern of regional abnormality underlying ineffective reappraisal performance across various diagnostic representations. However, the available meta‐analytic evidence at this point is not sufficient to draw such a conclusion due to the inconsistent results (McTeague et al., 2020; Picó‐Pérez, Radua, Steward, Menchón, & Soriano‐Mas, 2017; Wang et al., 2018). For example, although McTeague et al. (2020) found the right VLPFC as the only convergent region related to emotion dysregulation spanning different psychiatric diagnoses, this region was not identified in other meta‐analyses on a combination of mood and anxiety (Picó‐Pérez et al., 2017) and on a range of anxiety disorders (Wang et al., 2018). Divergent findings across these meta‐analyses encouraged us to address the dispute of transdiagnostic disruptions underlying reappraisal by performing a more comprehensive meta‐analysis on the largest available number of clinical neuroimaging studies concerning brain activation during a reappraisal task. In order to do so, we used the activation likelihood estimation (ALE) technique (Eickhoff et al., 2016; Eickhoff, Bzdok, Laird, Kurth, & Fox, 2012) to integrate the neuroimaging results. In particular, we performed the analyses in a hierarchical order (global level, experimental‐contrast level, and disorder‐group level) to map the neural correlates of reappraisal disruptions based on the increasing homogeneity of data. In this regard, we first pooled all the available data to provide an overview of the neural alterations in patients compared with healthy controls. Then, we clustered data by the factors with a potential to contribute to heterogeneity (i.e., regulation direction, stimulus valence, and instruction rule). Finally, we used a narrowing down approach to make clustering of disorders (from the most heterogeneous to the most homogenous ones) across the data representing downregulation of negative emotions.

## 2 METHODS AND MATERIALS

The current study was pre‐registered at the International Prospective Register of Systematic Reviews (PROSPERO, CRD42019119121) and the search strategy was based on the Preferred Reporting Items for Systematic Reviews and Meta‐Analyses statement (Moher, Liberati, Tetzlaff, & Altman, 2010). Following the recent best‐practice guidelines for neuroimaging meta‐analyses (Müller et al., 2018; Tahmasian et al., 2019), we performed several ALE analyses on the existing reappraisal neuroimaging studies that assessed the regulating disturbances in psychiatric patients compared with healthy controls. In a typical reappraisal experiment, participants are presented with a series of evocative stimuli and are instructed to either naturally respond to them or apply the reappraisal strategy by implementing a given tactic or choosing the tactic themselves. Generally, two specific tactics are used in reappraisal experiments: reinterpretation or changing one's reinterpretation of the emotional stimulus; and distancing or changing the one's psychological distance from the emotional stimulus (Webb et al., 2012). Reappraisal performance is assessed by contrasting the brain activations during the two conditions of reappraisal implementation and natural responding across individual studies.

### 2.1 Search strategies and selection criteria

The literature search was conducted in February 2020 through PubMed, ScienceDirect, and Web of Science databases with the following search terms: (cognitive OR volitional OR voluntary OR effortful) AND (emotion OR affect) AND (regulat* OR reappraisal OR reinterpretation OR distancing) AND (fMRI OR “functional MRI” OR “functional magnetic resonance imaging” OR PET OR “positron emission tomography”) AND (patient OR disorder). Additional publications were identified by reference tracking from reviews/meta‐analyses. The resulting pool consisted of 1,608 records, assessed by two authors independently (T.K. and Z.S.). Eligible studies were selected in two steps: Firstly, non‐English language publications, case reports, letters to editors, reviews/meta‐analyses, and structural or task‐free imaging studies were excluded by screening the abstracts. Secondly, full‐texts of all remaining studies were screened carefully and studies that met the following criteria were included:

- functional neuroimaging studies (i.e., fMRI/PET) that compared the reappraisal task between patients suffering from any kind of psychiatric disorders and healthy subjects,
- if significant brain activation results were reported for the contrast of interest (reappraisal vs. natural responding),
- if “whole‐brain” analyses were performed and coordinates from the peak of task‐based activations were reported in Montreal Neurological Institute (MNI) or Talairach spaces,
- if standardized diagnostic criteria (DSM‐IV‐TR, or DSM‐5, or ICD‐10) for patient recruitment was used,
- if adult subjects were recruited (range age between 18 and 60),
- if at least seven subjects were in each group.

### 2.2 Data extraction

For all the included studies, sample size, demographic data of participants (age, gender), clinical characteristics of patients (diagnosis, diagnostic tool, symptom severity, medication status, and comorbidities), experimental setup (stimulus arousal, stimulus valence, regulation strategy and regulation direction, imaging modality, scanner type, analysis software package, and statistical analysis criteria), and the peak coordinates of between‐group experiments for the reappraisal versus natural responding contrast were extracted (Table 1). In the present meta‐analysis, “study” reflects an individual publication and “experiment” indicates a set of coordinates belonging to a particular analysis or contrast of interest (i.e., patients vs. controls group comparison) in a given study (Müller et al., 2018). Subsequently, the experiments were coded as “increased” when the brain activation during the reappraisal condition was higher in patients than healthy controls (patients > controls) or “decreased” when it was lower in patients than controls (patients < controls). The peak coordinates reported in Talairach space were converted into MNI space to set all the coordinates in the same reference space (Lancaster et al., 2007). To avoid convergence over the analyses performed on the same/overlapping samples (reported within or between studies), we merged the coordinates from multiple experiments (e.g., increased/decreased) pertaining to the studies with the same/overlapping samples, making sure each study contributes once per analysis (Turkeltaub et al., 2012).

**TABLE 1:** Characteristics of 32 included studies in the present meta‐analysis

Study	Number (M:F)		Mean age (SD)		Diagnosis	Medication (*n*)	Comorbidity (*n*)	Symptom severity	ER tactic/direction	Stimulus	Arousal/valence	Statistical threshold	Software	MRI
	Patients	Controls	Patients	Controls										
*Major depressive disorder (MDD)*														
Stephanou, Davey, Kerestes, Whittle, and Harrison (2017)	53 (22:31)	64 (24:40)	19.72 (2.68)	19.03 (2.45)	SCID‐IV	No (within last 4 weeks)	No (lifetime)	MADRS [MDD = 32.80 (4.80); controls = 2.14 (2.99)]	Reinterpretation/downregulation	IAPS (negative social scenes), EPS, online sources	NA	FDR	SPM 8	3 T
Wang, Zhou, Dai, Ji, and Feng (2017)	12 (5:7)	15 (7: 8)	29.50 (8.46)	25.80 (5.89)	SCID‐IV	No (within last 2 weeks)	No (current psychiatric and lifetime neurologic)	BDI [MDD = 26.17 (12.65); controls = 4.27 (4.23)]	Not‐specified/downregulation and upregulation	IAPS (positive and negative images), other sources	NA	FWE (REST, AlphaSim)	SPM 8	3 T
Radke et al. (2018)	22 (13:9)	22 (13:9)	32.6 (10.9)	34.5 (9.9)	SCID‐IV	15	3	BDI [MDD = 13.8 (9.5); controls = 2.7 (3.4)]	Distancing/upregulation	FACES (angry face)	NA	FWE	SPM 8	3 T
Greening, Osuch, Williamson, and Mitchell (2014)	19 (6:13)	19 (6:13)	26.79 (11.4)	27.63 (11.0)	SCID‐IV	9	7	BDI [MDD = 25.53 (10.4); controls = 1.6 (2.3)]	Reinterpretation/downregulation	IAPS (sad and positive scenes)	Normative mean arousal sad = 5.08 (.62); mean arousal positive = 5.03 (.55)	FWE (AlphaSim)	AFNI 2012	3 T
Smoski, Keng, Schiller, Minkel, and Dichter (2013)	18 (4:15)	19 (7:12)	24.5 (5.4)	27.9 (6.3)	SCID‐IV	5	No (current)	BDI [MDD = 2.9 (5.0); controls = 1.4 (2.4)]	Not‐specified/downregulation	IAPS (sad pictures), other normed images	NA	FWE (AFNI, 3dClustSim)	FSL	3 T
Heller et al. (2009)	27 (12:15)	19 (9:10)	31.48 (11.58)	31.84 (14.65)	DSM‐IV	No (lifetime)	No (current psychiatric and lifetime bipolar/anxiety)	HAMD [MDD = 20.6 (2.39); controls = 1.2 (1.6)]	Not‐specified/downregulation and upregulation	IAPS (positive and negative pictures)	Normative mean valence negative = 2.95 (.87); mean arousal negative = 5.44 (.8); mean valence positive = 7.13 (.62); mean arousal positive = 5.28 (.58)	FWE (AlphaSim)	AFNI	3 T
Sheline et al. (2009)	24 (12:12)	21 (6:15)	34 (9.4)	35 (7.3)	DSM‐IV	No (within last 4 weeks)	No (lifetime)	HAMD [MDD = 21 (3.5); controls = 0 (.04)]	Not‐specified/downregulation	IAPS (positive and negative pictures)	NA	NA	NA	3 T
Johnstone, van Reekum, Urry, Kalin, and Davidson (2007)	21 (8:13)	28 (7:21)	33 (12)	28 (12)	DSM‐IV	No (current)	No (current psychiatric and lifetime bipolar)	HAMD [MDD = 21 (2.5); controls = .5 (.6)]	Not‐specified/downregulation and upregulation	IAPS (positive and negative pictures)	Normative mean valence negative = 2.95 (.87); mean arousal negative = 5.44 (.8); mean valence positive = 7.13 (.62); mean arousal positive = 5.28 (.58)	FWE (AlphaSim)	AFNI	3 T
*Schizophrenia*														
Zhang et al. (2020)	16 (12:4)	15 (10:5)	31.75 (8.7)	33.60 (11.1)	DSM‐IV and ICD‐10	NA	No (current)	PANSS [schizophrenia = 26.69 (7.1)]	Reinterpretation/downregulation	IAPS (negative pictures)	NA	FWE (AFNI 2018, 3dClustSim)	SPM 12	3 T
Larabi, van der Meer, Pijnenborg, Ćurčić‐Blake, and Aleman (2018)	30 (22:8)	15 (10:5)	35 (10.16)	33.6 (11.11)	DSM‐IV and ICD‐1O	28	No (current)	PANSS [schizophrenia = 57.9 (14.71)]	Reinterpretation/downregulation	IAPS (negative images)	Normative mean valence negative = 2.6; mean arousal negative = 5.7; mean valence neutral = 1.3; mean arousal neutral = 1.9	FWE	SPM 12	3 T
van der Meer et al. (2014)	20 (14:6)	20 (16:4)	35.5 (11.7)	35.2 (10.8)	DSM‐IV and ICD‐10	20	NA	PANSS [schizophrenia = 29.9 (7.7)]	Reinterpretation/downregulation	IAPS (negative images)	Normative mean valence negative = 2.6; mean arousal negative = 5.7; mean valence neutral = 1.3; mean arousal neutral = 1.9	FWE	SPM 5	3 T
Morris, Sparks, Mitchell, Weickert, and Green (2012)	12 (8:4)	15 (6:9)	44 (3)	35 (2)	DSM‐IV	12	NA	PANSS [schizophrenia = 32 (2)]	Distancing/downregulation and upregulation	IAPS (negative threat and suffering images)	NA	FWE	SPM 8	3 T
*Social anxiety disorder (SAD)*														
Ziv, Goldin, Jazaieri, Hahn, and Gross (2013)	27 (15:12)	27 (14:13)	31.1 (7.6)	32.6 (9.5)	DSM‐IV	No (current)	8	LSAS‐SR [SAD = 99.3 (11.8); controls = 15.3 (9.1)]	Reinterpretation/downregulation	Anger and contempt facial expressions	NA	FWE (AlphaSim)	AFNI	3 T
Goldin, Manber, Hakimi, Canli, and Gross (2009)	15 (6:9)	17 (8:9)	31.6 (9.7)	32.1 (9.3)	DSM‐IV	No (current)	No (current psychiatric and lifetime neurologic)	LSAS‐SR [SAD = 67.6 (21.1); controls = 29.3 (20.9)]	Distancing/downregulation	Facial action coding system (angry and contempt facial expression), and physical threat scenes	NA	FWE (AlphaSim)	AFNI	3 T
Goldin, Manber‐Ball, Werner, Heimberg, and Gross (2009)	27 (15:12)	27 (15:12)	32.1 (9.2)	32.2 (9.5)	DSM‐IV	No (current)	6	LSAS‐SR [SAD = 80.1 (16.8); controls = 15.7 (8.7)]	Reinterpretation/downregulation	Written social situations	NA	FWE (AlphaSim)	AFNI	3 T
*Posttraumatic stress disorder (PTSD)*														
Butler et al. (2018)	18 (18:0)	27 (27:0)	28.3 (6.4)	32.7 (5.9)	ICD‐10	No (current)	No (lifetime axis‐II)	PDS [PTSD = 36.28 (10.65); controls = 15.7 (8.7)]	Reinterpretation/downregulation	Combat images	NA	FWE (AFNI 2016, 3dClustSim)	SPM 8	3 T
Xiong et al. (2013)	20 (13:7)	20 (14:6)	32.92 (8.48)	31.53 (7.43)	SCID‐IV	No (lifetime)	No (lifetime, except of past depression)	CAPS [PTSD = 52.33 (9.44); controls = 8.26 (9.31)]	Reinterpretation/downregulation and upregulation	IAPS (negative images)	Normative mean valence negative = 2.17 (.34); mean arousal negative = 6.23 (.26); mean valence neutral = 5.12 (1.04); mean arousal neutral = 4.18 (.72)	FWE (REST, AlphaSim)	SPM 8	3 T
New et al. (2009)	14 (0:14)	14 (0:14)	38.7 (11.2)	31.7 (10.3)	SCID‐IV	No (lifetime)	No (lifetime, except of past depression)	CAPS [PTSD = 69.1 (17.6)]	Reinterpretation/downregulation and upregulation	IAPS (negative images)	NA	FWE (REST, AlphaSim)	SPM 2	3 T
*Bipolar disorder (BD)*														
Zhang et al. (2020)	15 (6:9)	15 (10:5)	39.87 (12.5)	33.60 (11.1)	DSM‐IV and ICD‐10	NA	No (current)	YMRS [BD = 1.4 (1.5)] and QIDS [BD = 5.27 (5.4)]	Reinterpretation/downregulation	IAPS (negative pictures)	NA	FWE (AFNI 2018, 3dClustSim)	SPM 12	3 T
Townsend et al. (2013)	30 (19:11)	26 (15:11)	37.9 (12.6)	35.5 (12.4)	SCID‐IV	21	No (current psychiatric and lifetime substance use/abuse)	YMRS [BD = 1.7 (2.2)] and HAMD [BD = 3.8 (1.9)]	Reinterpretation/downregulation	IAPS (negative images)	Normative mean valence negative = 2.8; mean arousal negative = 6.5	NA	FSL	3 T
Morris et al. (2012)	13 (8:5)	15 (6:9)	41 (3)	35 (2)	DSM‐IV	13	NA	NA	Distancing/downregulation	IAPS (negative threat and suffering images)	NA	FWE	SPM 8	3 T
*Borderline personality disorder (BPD)*														
van Zutphen et al. (2018)	55 (0:55)	42 (0:42)	30.88 (8.78)	28.33 (10.50)	SCID‐IV	55	49	BPD checklist [BPD = 120.6 (26.92); controls = 50.73 (5.03)]	Reinterpretation/downregulation and upregulation	IAPS (negative and positive), additional erotic pictures	NA	FWE	BrainVoyager	3 T
Schulze et al. (2011)	15 (0:15)	15 (0:15)	27.60 (7.85)	24.53 (2.85)	SCID‐IV	NA	7	BSL [BPD = 183.87 (53.64); controls = 49.60 (16.04)]	Distancing/downregulation and upregulation	IAPS (negative threat and suffering images)	NA	FWE	SPM 5	1.5 T
Koenigsberg et al. (2009)	18 (8:10)	16 (9:9)	32.6 (10.4)	31.8 (7.7)	SCID‐IV	No (within last 4 weeks)	No (lifetime)	ALS [BPD = 94.9 (23.7); controls = 20.3 (16.0)]	Distancing/downregulation	IAPS (interpersonal situations)	Normative mean valence negative = 2.35; mean arousal negative = 5.9; mean valence neutral = 5.2; mean arousal neutral = 3.65	FWE (REST, AlphaSim)	SPM 2	3 T
*Miscellaneous anxiety disorders*														
Blair et al. (2012)	53 (17:36)	18 (8:10)	33.73 (9.99)	33.4 (9.65)	SCID‐IV	No (within last 6 months)	No (current)	BAI [miscellaneous = 10.93 (7.17); controls = 2.3 (2.02)]	Reinterpretation/downregulation	IAPS (positive and negative images)	Normative mean valence negative = 3.08; mean arousal negative = 5.43; mean valence positive = 7.21; mean arousal positive = 5.15	FWE (AlphaSim)	AFNI	1.5 T
Ball, Ramsawh, Campbell‐Sills, Paulus, and Stein (2013)	41 (9:32)	22 (11:11)	32 (9)	27 (9)	DSM‐IV	No (within last 2 weeks)	17	QASIS [miscellaneous = 8.6 (3.3); controls = .8 (1.2)]	Not‐specified/downregulation	IAPS (negative images)	NA	FWE (3dClustSim)	AFNI	3 T
Campbell‐Sills et al. (2011)	13 (2:11)	13 (2:11)	NA	NA	SCID‐IV	No (lifetime)	12	NA	Reinterpretation/downregulation	IAPS (negative images)	NA	FWE (AlphaSim)	AFNI	3 T
*Panic disorder (PD)*														
Reinecke et al. (2015)	18 (4:14)	18 (4:14)	36.5 (13.8)	32.3 (12.1)	SCID‐IV	3	13	HADS‐anxiety [PD = 14.6 (4.1); controls = 2.0 (1.6)]	Reinterpretation/downregulation	IAPS (accidents or funerals)	Normative mean valence negative = 2.8 (1.7); mean arousal negative = 6.0 (2.2)	NA	FSL	3 T
*Pre‐menstrual dysphoric disorder (PMDD)*														
Petersen et al. (2018)	18 (0:18)	18 (0:18)	29.2 (7.24)	25.4 (6.99)	SCID‐IV	No (current)	No (lifetime except of unipolar mood disorders)	DRSP [PMDD = 3.53 (.63); controls = 1.01 (.05)]	Distancing/downregulation	IAPS (negative images) and other ones	NA	NA	FSL	3 T
*Obsession‐compulsion disorder (OCD)*														
Thorsen et al. (2019)	43 (21:22)	38 (18:20)	37.58 (10)	39.05 (11.27)	SCID‐IV	No (within last 4 weeks)	29	OCI‐R [OCD = 24.67 (11.79); controls = 3.37 (4.71)]	Not‐specified/downregulation	Fearful and OCD‐related pictures	NA	NA	SPM 8	3 T
*Substance use disorder (SUD)*														
Albein‐Urios et al. (2014)	17 (16:1)	18 (17:1)	36.41 (5.99)	30.50 (4.64)	SCID‐IV	NA	NO (current psychiatric and lifetime neurologic)	UPPS‐negative urgency [SUD = 33.17 (6.51); controls = 22.22 (5.1)]	Not‐specified/downregulation	IAPS (negative images)	Normative mean valence negative = 3.51 (.86); mean arousal negative = 5.70 (0.6)	FDR	SPM 8	3 T
*Gambling disorder (GD)*														
Navas et al. (2017)	17 (16:1)	21 (20:1)	32.94 (7.77)	31 (4.6)	SCID‐IV	NA	No (current psychiatric and lifetime neurologic)	UPPS‐negative urgency [GD = 29.18 (4.7); controls = 23.19 (5.46)]	Reinterpretation/downregulation	IAPS (negative mutilation pictures)	Normative mean valence negative = 3.51 (.86); mean arousal negative = 5.70 (0.6)	FWE (REST, AlphaSim)	SPM 8	3 T

Abbreviations: ALS, Affect Lability Scale; BAI, Beck Anxiety Inventory; BDI, Beck Depression Inventory; BSL, Borderline Symptom List; CAPS, Clinician‐Administered PTSD Scale; DRSP, Daily Record of Severity of Problems; HADS, Hospital Anxiety and Depression Scale; HAMD, Hamilton Depression Rating Scale; LSAS‐SR, Liebowitz Social Anxiety Scale—Self‐Report; MADRS, Montgomery–Asberg Depression Rating Scale; OCI‐R, Obsessive Compulsive Inventory‐Revised; PANASS, Positive and Negative Syndrome Scale; PDS, Posttraumatic Diagnostic Scale; QASIS, Overall Anxiety Severity and Impairment Scale; QIDS, Quick Inventory of Depressive Symptomatology; UPPS, urgency, premeditation, perseverance, and sensation seeking scale; YMRS, Young Mania Rating Scale.

### 2.3 Activation likelihood estimation

A revised version of ALE (Eickhoff et al., 2012) was used to assess whether the activation foci reported in peak coordinates significantly clustered into specific spatial locations, rather than randomly distributed across the whole brain. The ALE analyses were performed in three steps: First, spatial 3D Gaussian probability distributions were modeled around the peak coordinates of activated foci from experiments of interest. The width of the aforementioned probability determines the spatial uncertainty associated with variations in sampling effects, data processing, and data analysis. Since the foci of contrasts with smaller sample size have a smaller effect on the modeled uncertainty, it was adjusted for the number of subjects in the smaller group. Then, “modeled activation” maps of all foci from each experiment were pooled into an ALE activation map by computing their overlap across the experiments (Eickhoff et al., 2012; Turkeltaub et al., 2012). Finally, the ALE maps were assessed against a null distribution map to enable the random‐effects inference by using nonlinear histogram integration (Eickhoff et al., 2012; Turkeltaub et al., 2012). As suggested previously, the above‐chance clustering activation foci were assessed by setting the threshold at *p* < .05 family‐wise error at the cluster level (cFWE) to correct for multiple comparisons and avoid spurious findings.

To identify potential transdiagnostic patterns of disturbed recruitments of neural mechanisms responsible for reappraisal, we conducted a set of complementary ALE analyses at three hierarchical levels based on the homogeneity of experiments: global level, experimental‐contrast level, and disorder‐group level (see Table 2). At the global level, we assessed convergence across all reported effects by pooling all experiments, and further, analyzed increased/decreased activations separately. At the experimental‐contrast level, we first performed three independent analyses on the regulation goal (downregulation/upregulation), stimulus valence (negative/positive), and reappraisal instruction (reinterpretation/distancing/not‐specified). Notably, a valid analysis was only possible for “downregulation” and “negative” as there were not enough experiments in the other groups of data (<17) (Eickhoff et al., 2016). Then, we restricted the analysis to the experiments that reported peak coordinates for “downregulation” and “negative valence” simultaneously (i.e., negative downregulation). At the disorder‐group level, we performed the analyses on the negative downregulation experiments to explore the effects of increasing disorder homogeneity on the nature of disturbances, while patients were applying reappraisal to downregulate their negative emotions. In this regard, we used a stepwise narrowing down approach to group the experiments. For the first step, we restricted the experiments to nonpsychotic disorders by excluding the ones belonging to schizophrenia. For the second step, we restricted the experiments of nonpsychotic disorders to those belonging to emotional disorders which are identified with emotional disturbances as their hallmark (i.e., borderline personality as well as mood and anxiety disorders; Bullis, Boettcher, Sauer‐Zavala, Farchione, & Barlow, 2019). Finally, for the third step, we restricted the experiments of emotional disorders to those belonging to the disorders with shared neural phenotypes (i.e., mood and anxiety disorders; Janiri et al., 2020). No other sets of experiments (i.e., specific category or type of disorders) had enough data for a valid ALE analysis.

**TABLE 2:** Findings of conducted meta‐analyses in patients compared with healthy subjects

ALE analysis	Experiments	Contrast	*p*‐value		Number of experiments
			TFCE	cFWE	
Global level	Pooled	All	.406	.418	28
	Decreased	Patients < controls	.436	.570	21
	Increased	Patients > controls	.675	.832	20
Experimental‐contrast level	Downregulation	All	.662	.859	27
	Negative	All	.615	.930	28
	Negative downregulation	All	.759	.850	27
Disorder‐group level	1. Across nonpsychotic disorders	All	.632	.751	25
	2. Across emotional disorders	All	.889	.859	24
	3. Across mood and anxiety disorders	All	.658	.655	21

Abbreviations: ALE, activation likelihood estimation; cFWE, cluster‐level family‐wise error; TFCE, threshold‐free cluster enhancement.

## 3 RESULTS

**FIGURE 1:**
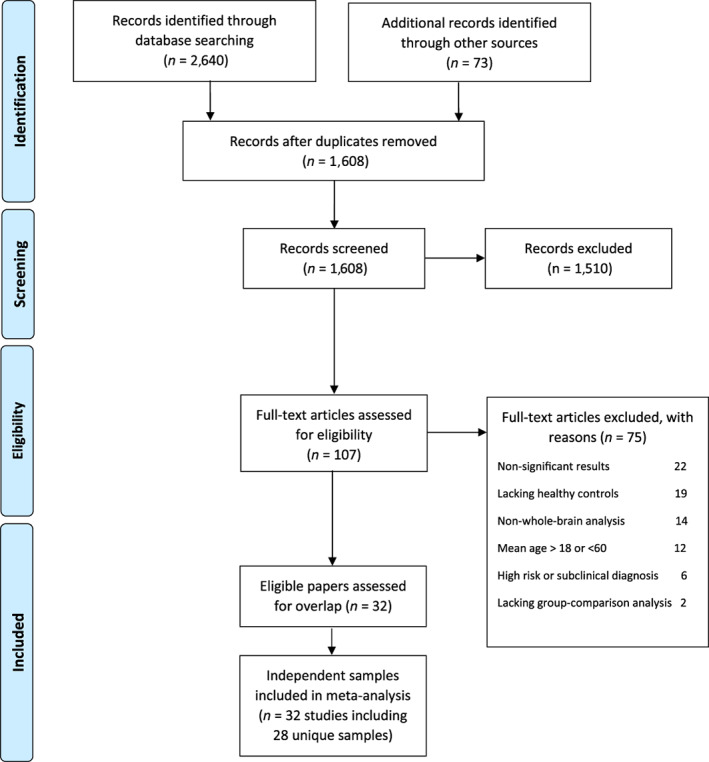
Study selection strategy flow chart

A pool of 1,608 records was screened and 107 full‐text publications were assessed for eligibility. Subsequently, 75 studies were excluded for the following reasons: lacking either healthy controls or group comparison analyses, restricting samples to adolescents or older adults, not performing whole‐brain analysis, not reporting significant group comparison results, and including individuals without DSM/ICD‐based diagnosis (e.g., at a high risk of mental illnesses or with subclinical conditions) (Table S1). Finally, 32 publications were included in our meta‐analysis (Figure 1), with three overlapping samples reported in multiple publications, one sample used in three articles (Larabi et al., 2018; van der Meer et al., 2014; Zhang et al., 2020), and two samples each used in two articles (Goldin, Manber‐Ball, et al., 2009; Heller et al., 2009; Johnstone et al., 2007; Ziv et al., 2013). As mentioned earlier, we rigorously avoided including the same/overlapping samples within and across articles. Accordingly, we merged all studies with overlapping samples that resulted in 28 independent samples. Demographic, clinical, and experimental characteristics of the included articles are shown in Table 1. Overall, we conducted several ALE meta‐analyses (Table 2).

**FIGURE 2:**
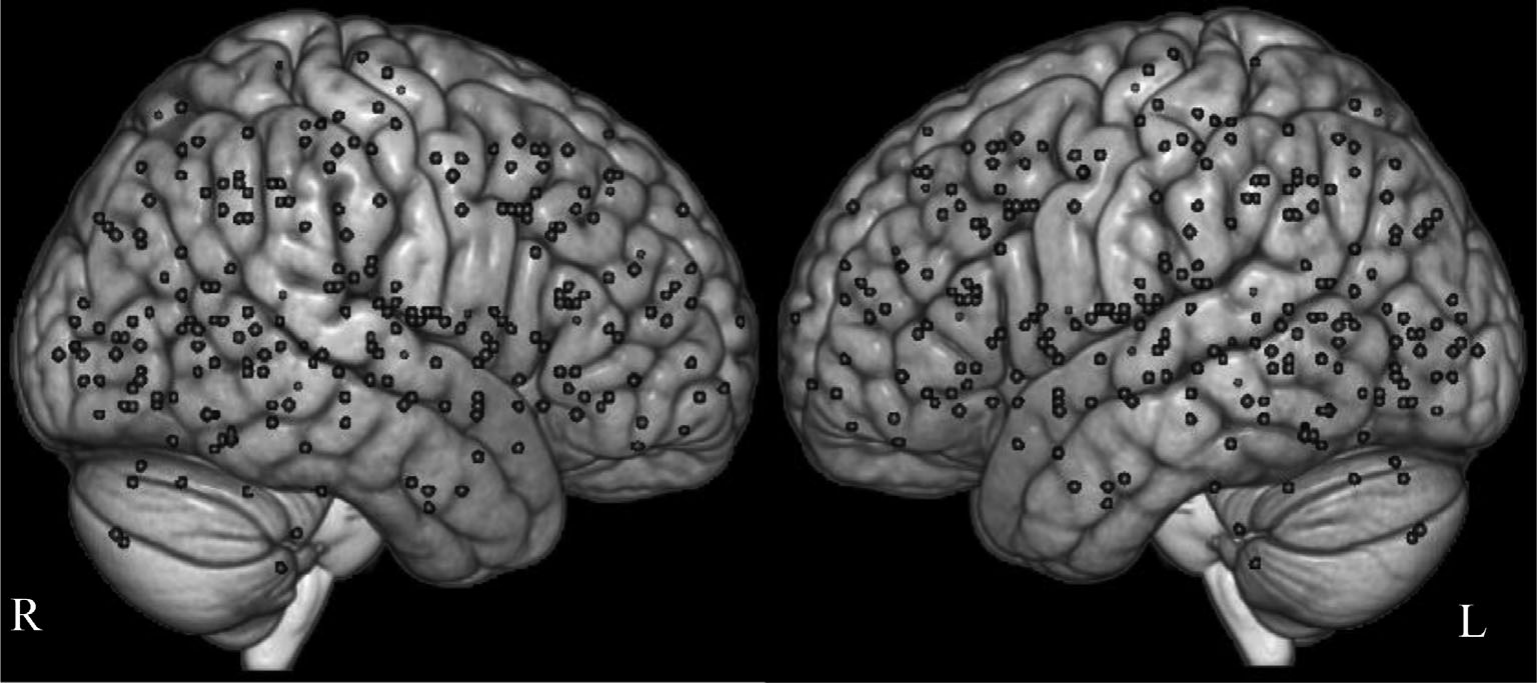
Distribution of the included peak coordinates in the current study. The represented foci reflect functional alterations related to the reappraisal task in patients with various psychiatric disorders compared with healthy subjects

Surprisingly, none of our global level, experimental‐contrast level or disorder‐group level analyses yielded significant results:

- global level analyses included [“pooled”: 28 experiments (*p* = .418), “decreased”: 21 experiments (*p* = .570) and “increased”: 20 experiments (*p* = .832)],
- experimental‐contrast level analyses included [“downregulation”: 27 experiments (*p* = .859), “negative”: 28 experiments (*p* = .930), “negative downregulation”: 27 experiments (*p* = .850)],
- disorder‐group level analyses included [“nonpsychotic disorders”: 25 experiments (*p* = .751), “emotional disorders”: 24 experiments (*p* = .859), “mood and anxiety disorders”: 21 experiments (*p* = .655)].
  Of note, repeating all analyses with a more liberal statistical threshold (i.e., threshold‐free cluster enhancement, TFCE) demonstrated no significant convergence either (Table 2). Figure 2 displays the sporadic distribution of foci across the included experiments.

## 4 DISCUSSION

In the current meta‐analysis, we explored whether there is a convergent regional brain abnormality related to dysfunctional reappraisal across various psychiatric disorders. Considering the homogeneity of included tasks (i.e., reappraisal) and using a strictly statistical approach for multiple comparison correction (i.e., cFWE), our ALE analyses did not yield any significant results, indicating the divergence of reappraisal impairments across different forms of psychopathology. Our results are in line with some previous meta‐analytic findings in patient populations that did not reveal spatial convergence of brain abnormalities (Degasperi, Cristea, Di Rosa, Costa, & Gentili, 2021; Giehl, Tahmasian, Eickhoff, & van Eimeren, 2019; Huang, Rootes‐Murdy, Bastidas, Nee, & Franklin, 2020; Müller et al., 2017; Nickl‐Jockschat, Janouschek, Eickhoff, & Eickhoff, 2015; Saberi, Mohammadi, Zarei, Eickhoff, & Tahmasian, 2021; Samea et al., 2019; Sheng et al., 2020; Tahmasian et al., 2018). This variance could be attributable to the complex physiological and pathophysiological mechanisms of reappraisal, heterogeneity in clinical populations, and/or experimental or methodological divergence (Tahmasian et al., 2018). We further discussed this heterogeneity as follows.

### 4.1 Distinct pathophysiology of impaired reappraisal across psychiatric disorders

#### 4.1.1 Cognitive view

Theoretically, emotion dysregulation may take place in different stages including the identification of regulation necessity, selection of regulatory strategy, implementation of selected strategy, and stopping/switching of the implemented process (Fernandez et al., 2016; Gross, Uusberg, & Uusberg, 2019). Indeed, clinical conditions can be characterized by cognitive impairments in different regulatory stages and may not involve disruptions in common brain regions. For example, major depressive disorder is involved with overestimation of mood‐congruent stimuli, (Zilverstand et al., 2017) and conversely, helplessness to ignite a regulatory action (Sheppes, Suri, & Gross, 2015). Patients with bipolar disorder overvalue the hedonic benefits of manic states and are not usually convinced to downregulate the positive affect (Fernandez et al., 2016). Anxiety is associated with attentional biases toward threat stimuli, (Zilverstand et al., 2017) and consequently an amplified representation of regulation urgency (Gross & Jazaieri, 2014). An exaggerated sense of regulation necessity and decreased flexibility in strategy selection are mainly observed in posttraumatic stress disorder and obsessive–compulsive disorder (Taylor & Liberzon, 2007). Patients with social anxiety disorder may be uncertain about the effectiveness of reappraisal implementation due to insufficient self‐efficacy, which probably results in premature stopping of regulatory effort (Sheppes et al., 2015). Borderline personality disorder is associated with monitoring deficits related to impulsive strategy switching (Gross et al., 2019). And finally, failing to stop maladaptive strategies (e.g., rumination) in depressive and anxiety disorders may affect the implementation of adaptive strategies including reappraisal (Dryman & Heimberg, 2018). All these examples show that differences in clinical populations regarding the awareness of emotions, beliefs about the controllability of emotions, tendency to regulate emotions, and availability of regulatory resources (Kim, Bigman, & Tamir, 2015) are critical factors influencing the successful reappraisal performance.

#### 4.1.2 Neurobiological view

Abnormal reappraisal in individuals with psychopathology generally occurs in the form of disrupted top‐down modulation of emotion processing (Zilverstand et al., 2017). However, the exact neurophysiological patterns may vary across specific disorders. In fact, the intimate connection between emotion generation and emotion regulation systems (Ochsner et al., 2004) can make it difficult to determine the extent to which the cross‐disorder regulation impairments can be explained in terms of common pathways. In other words, cognitive regulatory mechanisms are thought to rely on a set of cortical–cortical and cortical–subcortical networks (Morawetz et al., 2020; Sripada et al., 2014) that facilitate top‐down modulation of emotions across hierarchical levels of emotion processing (Smith & Lane, 2015). Thus, it is plausible that distinct disturbances in different hierarchical networks may lead to emotion dysregulation in the form of reappraisal impairments. For example, in anxiety disorders, excessive activation in the amygdala during the appraisal of aversive stimuli may result in the generation of intensive emotions, which can challenge the regulation system (Brehl, Kohn, Schene, & Fernández, 2020; Silvers et al., 2014). On the other hand, atypical recruitment of prefrontal regulatory networks may be the underlying cause of dysfunctional reappraisal among patients with schizophrenia or bipolar disorder (Silvers et al., 2014; Tully & Niendam, 2014). Abnormal network interactions between the prefrontal and subcortical structures may be another source of regulation disruption (Berboth & Morawetz, 2021). For instance, poor top‐down regulation in major depressive disorder can be recognized with a diminished negative correlation between the amygdala and prefrontal cortices (Park et al., 2019). Collectively, these examples indicate that ineffective reappraisal in psychopathology might result from disturbances at different levels of emotional processing rather than just a particular regional abnormality.

### 4.2 Experimental task design issues

The absence of convergent regional abnormality due to the reappraisal impairment can be further explained by the taxonomy of experimental designs in neuroimaging studies of reappraisal. Although we only included studies that used the prototypical reappraisal paradigm, some experimental factors and underlying cognitive functions could well affect the neural basis of cognitive reappraisal. Attentional engagement is a relevant example that can be modulated with interrelated exogenous factors such as arousal and valence (Sussman, Heller, Miller, & Mohanty, 2013) and endogenous factors like needs, goals, and motivations (Ochsner & Gross, 2005). Remarkably, reappraising high arousal stimuli involves greater cognitive demands (Ortner, Ste Marie, & Corno, 2016), as appeared in differential prefrontal recruitment (Silvers, Weber, Wager, & Ochsner, 2015). Relatedly, various negative stimuli (e.g., sad, disgusting, and fearful) are reported to reflect similar arousals, but involve different emotion processing networks (Fusar‐Poli et al., 2009; Vytal & Hamann, 2010), suggesting their potentially distinct regulatory circuitries. In particular, disorder‐relevant stimuli are expected to be more salient than irrelevant ones (Hagemann, Straube, & Schulz, 2016). Thus, at least a part of nonreplicable results may stem from uncontrolled moderating factors related to external heterogeneous stimuli and/or internal diverse representations.

Another important factor that may not be well indexed by prototypical reappraisal tasks is the temporal dynamics of reappraisal, which is thought to engage different neural substrates for implementing and maintaining new appraisals (Kalisch, 2009). In a standard reappraisal task, individuals are given instructions on how and when to regulate their emotions. This paradigm usually measures the capability of individuals to implement reappraisal‐related cognitive processes to regulate their emotions (Silvers & Moreira, 2019). However, their tendency to engage regulatory mechanisms without being instructed (Doré, Weber, & Ochsner, 2017) or their ability to keep and monitor reappraised images or thoughts (Kalisch, 2009) are not generally evaluated by these experiments. Therefore, by using a standard reappraisal paradigm, it is not possible to capture the difficulties psychiatric patients may encounter while self‐initiating the regulation process or maintaining the reframed emotional states after applying the reappraisal successfully.

### 4.3 Heterogeneity of demographic and clinical characteristics of psychiatric patients

In this meta‐analysis, we included adult patients aged between 18 and 60 years in order to exclude the potential effect of adolescent and aged brains on the neural correlates of reappraisal (Ahmed, Bittencourt‐Hewitt, & Sebastian, 2015; Lantrip & Huang, 2017; Nashiro, Sakaki, & Mather, 2012). However, emotion regulation is a dynamic process and may change across the lifespan (Consedine & Mauss, 2014; Livingstone & Isaacowitz, 2021). Thus, a part of the neural heterogeneity can be explained by the fact that inevitable brain changes may occur across different ages even in our restricted age range (Allard & Kensinger, 2014). Other characteristics of patients such as gender (McRae, Ochsner, Mauss, Gabrieli, & Gross, 2008; Whittle, Yücel, Yap, & Allen, 2011), medication status (Roiser & Sahakian, 2013), and severity of their illness (Dixon et al., 2020; Stephanou et al., 2017) can also be confounders for our divergent findings (Table 1). Additionally, heterogeneity of psychiatric disorders (Feczko et al., 2019), which are expressed both across diagnostic criteria and underlying neural substrates can be another source of inconsistency. For example, major depressive disorder is a highly heterogeneous syndrome (Lynch, Gunning, & Liston, 2020) that presents itself in a number of variants with different somatic/emotional/cognitive and clinical states (Drysdale et al., 2017; Tokuda et al., 2018). Specifically, in the case of emotion dysregulation, various trends of neural disturbances have emerged across patients with depression (Rive et al., 2013; Silvers et al., 2014). Similarly, in other psychiatric disorders, such as generalized anxiety disorder and borderline personality disorder several patterns of atypical brain activation during reappraisal performance have been recognized (Silvers et al., 2014). Taken together, these findings suggest that heterogeneity in clinical or demographic characteristics of patients may importantly contribute to the inconsistent findings regarding the neural correlates of impaired reappraisal.

### 4.4 Flexible methodology and publication bias

Methodological flexibility in neuroimaging studies (e.g., image acquisition, preprocessing, and analysis pipeline; Bowring, Maumet, & Nichols, 2019; Masouleh, Eickhoff, Hoffstaedter, Genon, & Initiative, 2019) could also explain our null findings. The noticeable effects of analytical variability on the results of neuroimaging studies and their interpretation have been indicated in a recent neuroimaging study in which 70 independent teams analyzed the same dataset and even no two teams followed identical analysis workflows (Botvinik‐Nezer et al., 2020). As a relevant example in our meta‐analysis, the different approaches that are employed for multiple testing adjustment might be a potential reason for our nonreplicable findings (Table 1).

In addition to the lack of a validated analytical workflow, positive‐results bias or tendency to publish significant findings is another potential explanation for our nonreplicable results (Jennings & van Horn, 2012). Moreover, when nonsignificant results are published, they are not entered in ALE meta‐analyses (Müller et al., 2018). So, the insensitivity of ALE to nonsignificant results increases the publication bias. Accordingly, we had to exclude 22 eligible studies because of their null findings (Table S1), despite knowing the importance of their valuable results (Mervis, 2014). Thus, at least in some cases, the identified reappraisal disturbances in clinical populations could have resulted from a biased overestimation. For example, regarding depression, there is evidence for the intact neural underpinning of reappraisal (Davis, Foland‐Ross, & Gotlib, 2018; Doré et al., 2018; Loeffler et al., 2019; Rubin‐Falcone et al., 2018) or at least effective implementation of reappraisal when patients are explicitly trained to do so (Ebneabbasi et al., 2021; Liu & Thompson, 2017). Hence, some of the observed heterogeneity in reappraisal literature might be due to methodological diversity or publication bias.

### 4.5 Collation with previous meta‐analyses

Following the best‐practice guideline for neuroimaging meta‐analysis (Müller et al., 2018; Tahmasian et al., 2019) and the rigorous methodological approach, our null result is expected to reflect a representation of the existing reappraisal studies across psychiatric disorders and should not be attributable to a lack of statistical power or methodological issues. Up to now, three meta‐analyses have been performed on the functional organization of reappraisal in psychiatrically ill populations (McTeague et al., 2020; Picó‐Pérez et al., 2017; Wang et al., 2018). Two of these meta‐analyses are conducted on specific categories of disorders including a combination of mood and anxiety disorders (Picó‐Pérez et al., 2017) with a higher proportion of mood disorders (9/4), and a range of anxiety disorders (Wang et al., 2018). Although both of these meta‐analyses have yielded convergence of brain abnormalities across their included studies, they do not indicate consistent findings compared with each other. The heterogeneity of their findings supports our null result, especially, when we restricted the analysis to only those experiments representing the merged category of mood and anxiety disorders (Table 2). However, our study has some differences compared with these meta‐analyses that make the comparison between the results difficult. Firstly, these meta‐analyses used the effect size signed differential mapping (ES‐SDM) method, which is statistically more lenient than ALE (Müller et al., 2018). Secondly, these studies performed the analyses on a low number of studies (13 and 11, respectively), and each of them included two nonsignificant studies. Thus, their findings of significant convergence might be driven by only a few experiments. Thirdly, due to the nature of ALE method, we did not have enough experiments to perform analysis on each category of mood and anxiety disorders (Table 2) to see if we could replicate their disorder‐specific findings. Lastly, our search was not restricted to mood and anxiety disorders, and thereby, additional relevant disorders were covered. The third transdiagnostic meta‐analysis (McTeague et al., 2020) was performed on a pool of 18 studies including patients with various psychiatric disorders. This study found a consistent brain abnormality located in the right ventrolateral prefrontal cortex. Despite adhering to the same analytic method (i.e., ALE), we did not replicate their result, indicating that by increasing the number of studies, as well as covering additional relevant disorders (i.e., borderline personality disorder, premenstrual depressive disorder, and gambling disorder), the obtained consistency was not observed anymore. Of note, having included two regulation studies other than reappraisal may have also influenced their study results. However, similar to our study they did not find significant results for the increased/decreased analyses, which may indicate the fragility of their finding for the pooled analysis. We additionally explored the role of regulation goal and stimulus valence both separately and in combination, as well as the effect of homogeneity of disorders by narrowing down the spectrum of included disorders in three subsequent steps to see where we can get transdiagnostic patterns of disturbances. Collectively, none of our complementary analyses yielded significant findings.

### 4.6 Limitations and recommendations for future studies

Our study has some limitations as well. First, the number of patients in the included studies differed substantially across the included psychiatric groups. The disproportionate share of coordinates, therefore, may affect the sensitivity of results and may lead to overemphasizing the larger diagnostic groups (e.g., major depressive disorder). Second, none of the particular diagnostic groups reached the minimum number of experiments to obtain sufficient power for ALE analysis, which forecloses further representation of their pathologically related differences in brain activation. And third, 22 eligible studies with nonsignificant group comparison results were excluded due to the insensitivity of ALE to nonsignificant results, which may have affected the robustness of our findings. These substantial limitations restrict the generalizability of our null findings and emphasize the need for further original studies on various psychiatric patients using larger sample sizes and standard unbiased methodologies, ideally through collaborations to ameliorate site idiosyncrasies, as well as sharing data openly to allow replication and future integration.

Furthermore, to differentiate a true lack of localized consistency from clinical/methodological divergence, we propose the following recommendations for future clinical neuroimaging studies: (a) investigate the neural correlates of model‐driven/stage‐based regulatory dysfunctions in clinical populations; (b) design the experiments considering the temporal dynamics of the reappraisal process (i.e., initiation, implementation, and maintenance), and experimental moderating factors such as stimulus features (e.g., valence, arousal, relevancy to disorder); (c) report clinical (e.g., comorbidity, medication, age/gender, symptom severity) as well as methodological (e.g., preprocessing, software and analysis pipeline) characteristics for replication feasibility; and, (d) utilize stringent statistical thresholds to minimize the potential nonreplicable spurious results. Moreover, we recommend future transdiagnostic meta‐analyses to use other techniques such as hierarchical clustering (Morawetz et al., 2020) or psychophysiological interaction (Berboth & Morawetz, 2021) analyses to investigate the regulation disruptions beyond regional disturbances, if enough experiments are available.

## 5 CONCLUSION

The present meta‐analysis demonstrated that the existing literature on emotion dysregulation has not yielded consistent, localized, and cross‐cutting neural abnormality during reappraisal performance. We highlighted the distinct psychopathology of impaired reappraisal across different clinical populations as well as divergent experimental, clinical, and methodological factors that could explain our null results. Our transdiagnostic neuroimaging meta‐analysis highlights the importance of simultaneous evaluation of psychiatric disorders in order to construct a multilevel understanding of neuropsychopathology (Barch, 2020; Fusar‐Poli et al., 2019). Even though a transdiagnostic approach generally helps to map the commonalities of psychiatric disorders (Goodkind et al., 2015; McTeague et al., 2017; McTeague et al., 2020; Sha, Wager, Mechelli, & He, 2019; Yaple, Tolomeo, & Rongjun, 2021; Zhang et al., 2016), our study underscores the complexity of studying the neural abnormalities related to higher‐order cognitive processes including reappraisal.

## CONFLICT OF INTERESTS

The authors declare that the research was conducted in the absence of any commercial or financial relationships that could be construed as a potential conflict of interest.

## Supporting information


**Appendix S1** Supporting Information.

## Data Availability

The data that support the findings of this study are available from the corresponding author upon reasonable request.
