# Supplementary material for: In search of convergent regional brain abnormality in cognitive emotion regulation: A transdiagnostic neuroimaging meta‐analysis
Source: Hum Brain Mapp. 2021 Nov 26;43(4):1309–25. doi: 10.1002/hbm.25722 (PMC8837597; doi:10.1002/hbm.25722)
Supplement: Supplementary file 1 — Appendix S1 Supporting Information. [file HBM-43-1309-s001.docx]

**Supplementary Table 1:** List of excluded studies with reasons**.**

| **STUDY** | **REASON OF EXCLUSION** |
| --- | --- |
| (Kreifelts, Brück et al. 2017) | Non-significant results |
| (Fitzgerald, MacNamara et al. 2017) | Non-significant results |
| (Corbalán, Beaulieu et al. 2015) | Non-significant results |
| (Gaebler, Daniels et al. 2014) | Non-significant results |
| (Dillon and Pizzagalli 2013) | Non-significant results |
| (Rubin-Falcone, Weber et al. 2020) | Non-significant results |
| (Dixon, Moodie et al. 2020) | Non-significant results |
| (Jansen, van den Heuvel et al. 2019) | Non-significant results |
| (Jansen, Van Den Heuvel et al. 2019) | Non-significant results |
| (Loeffler, Satterthwaite et al. 2019) | Non-significant results |
| (Chrysikou, Wing et al. 2019) | Non-significant results |
| (Materna, Wiesner et al. 2019) | Non-significant results |
| (Davis, Foland-Ross et al. 2018) | Non-significant results |
| (Doré, Rodrik et al. 2018) | Non-significant results |
| (Rubin-Falcone, Weber et al. 2018) | Non-significant results |
| (Denny, Fan et al. 2015) | Non-significant results |
| (Wang, Feng et al. 2014) | Non-significant results |
| (Albein-Urios, Verdejo-Román et al. 2013) | Non-significant results |
| (Fitzgerald, Phan et al. 2017) | Non-significant results |
| (Schmitt, Winter et al. 2016) | Non-significant results |
| (Light, Heller et al. 2011) | Non-significant results |
| (Yip, Gross et al. 2018) | Non-significant results |
| (Hilland, Landrø et al. 2018) | Lacking healthy controls |
| (Zhang, Dong et al. 2020) | Lacking healthy controls |
| (Contreras-Rodríguez, Albein-Urios et al. 2020) | Lacking healthy controls |
| (Fitzgerald, Klumpp et al. 2019) | Lacking healthy controls |
| (Schmitgen, Niedtfeld et al. 2019) | Lacking healthy controls |
| (Gorka, Young et al. 2019) | Lacking healthy controls |
| (Klumpp, Roberts et al. 2017) | Lacking healthy controls |
| (Fonzo, Goodkind et al. 2017) | Lacking healthy controls |
| (Fonzo, Goodkind et al. 2017) | Lacking healthy controls |
| (Klumpp, Fitzgerald et al. 2017) | Lacking healthy controls |
| (Reinecke, Thilo et al. 2018) | Lacking healthy controls |
| (Klumpp, Roberts et al. 2017) | Lacking healthy controls |
| (Goldin, Ziv et al. 2014) | Lacking healthy controls |
| (Reinecke, Thilo et al. 2014) | Lacking healthy controls |
| (Brühl, Herwig et al. 2013) | Lacking healthy controls |
| (Goldin, Ziv et al. 2013) | Lacking healthy controls |
| (Heller, Johnstone et al. 2013) | Lacking healthy controls |
| (Goldin, Ziv et al. 2013) | Lacking healthy controls |
| (Hermann, Schäfer et al. 2009) | Lacking healthy controls |
| (Kanske, Schönfelder et al. 2015) | Non-whole-brain analysis |
| (Rabinak, MacNamara et al. 2014) | Non-whole-brain analysis |
| (Kanske, Heissler et al. 2012) | Non-whole-brain analysis |
| (Lang, Kotchoubey et al. 2012) | Non-whole-brain analysis |
| (Erk, Mikschl et al. 2010) | Non-whole-brain analysis |
| (Douw, Quaak et al. 2020) | Non-whole-brain analysis |
| (Klumpp, Kinney et al. 2019) | non-whole-brain analysis |
| (Jacob, Shany et al. 2019) | Non-whole-brain analysis |
| (Young, LeBeau et al. 2019) | Non-whole-brain analysis |
| (Zhang, Opmeer et al. 2018) | Non-whole-brain analysis |
| (Rive, Mocking et al. 2015) | Non-whole-brain analysis |
| (De Wit, Van der Werf et al. 2015) | Non-whole-brain analysis |
| (Ball, Stein et al. 2014) | Non-whole-brain analysis |
| (Hermann, Leutgeb et al. 2013) | Non-whole-brain analysis |
| (Raschle, Fehlbaum et al. 2019) | Mean age ≥ 18 or ≤ 60 |
| (Seidel, King et al. 2018) | Mean age ≥ 18 or ≤ 60 |
| (Seidel, King et al. 2018) | Mean age ≥ 18 or ≤ 60 |
| (LeWinn, Strigo et al. 2018) | Mean age ≥ 18 or ≤ 60 |
| (Cisler, Sigel et al. 2016) | Mean age ≥ 18 or ≤ 60 |
| (Murphy, Barch et al. 2016) | Mean age ≥ 18 or ≤ 60 |
| (Belden, Pagliaccio et al. 2015) | Mean age ≥ 18 or ≤ 60 |
| (Platt, Campbell et al. 2015) | Mean age ≥ 18 or ≤ 60 |
| (Karim, Tudorascu et al. 2017) | Mean age ≥ 18 or ≤ 60 |
| (Pitskel, Bolling et al. 2014) | Mean age ≥ 18 or ≤ 60 |
| (Andreescu, Sheu et al. 2015) | Mean age ≥ 18 or ≤ 60 |
| (Perlman, Simmons et al. 2012) | Mean age ≥ 18 or ≤ 60 |
| (Meluken, Ottesen et al. 2019) | High risk or subclinical diagnosis |
| (Simsek, Oguz et al. 2017) | High risk or subclinical diagnosis |
| (Van Der Velde, Opmeer et al. 2015) | High risk or subclinical diagnosis |
| (Heissler, Kanske et al. 2014) | High risk or subclinical diagnosis |
| (Felder, Smoski et al. 2012) | High risk or subclinical diagnosis |
| (Modinos, Ormel et al. 2010) | High risk or subclinical diagnosis |
| (Walsh, Eisenlohr-Moul et al. 2019) | Lacking group-comparison analysis |
| (Anand, Grandhi et al. 2019) | Lacking group-comparison analysis |

Albein-Urios, N., J. Verdejo-Román, C. Soriano-Mas, S. Asensio, J. M. Martínez-González and A. Verdejo-García (2013). "Cocaine users with comorbid Cluster B personality disorders show dysfunctional brain activation and connectivity in the emotional regulation networks during negative emotion maintenance and reappraisal." European Neuropsychopharmacology **23**(12): 1698-1707.

Anand, A., J. Grandhi, H. Karne and J. M. Spielberg (2019). "Intrinsic functional connectivity during continuous maintenance and suppression of emotion in bipolar disorder." Brain Imaging and Behavior: 1-11.

Andreescu, C., L. K. Sheu, D. Tudorascu, J. J. Gross, S. Walker, L. Banihashemi and H. Aizenstein (2015). "Emotion reactivity and regulation in late-life generalized anxiety disorder: functional connectivity at baseline and post-treatment." The American Journal of Geriatric Psychiatry **23**(2): 200-214.

Ball, T. M., M. B. Stein, H. J. Ramsawh, L. Campbell-Sills and M. P. Paulus (2014). "Single-subject anxiety treatment outcome prediction using functional neuroimaging." Neuropsychopharmacology **39**(5): 1254-1261.

Belden, A. C., D. Pagliaccio, E. R. Murphy, J. L. Luby and D. M. Barch (2015). "Neural activation during cognitive emotion regulation in previously depressed compared to healthy children: Evidence of specific alterations." Journal of the American Academy of Child & Adolescent Psychiatry **54**(9): 771-781.

Brühl, A. B., U. Herwig, A. Delsignore, L. Jäncke and M. Rufer (2013). "General emotion processing in social anxiety disorder: neural issues of cognitive control." Psychiatry Research: Neuroimaging **212**(2): 108-115.

Chrysikou, E. G., E. K. Wing and W. O. van Dam (2019). "Transcranial Direct Current Stimulation Over Prefrontal Cortex in Depression Modulates Cortical Excitability in Emotion Regulation Regions as Measured by Concurrent Functional Magnetic Resonance Imaging: An Exploratory Study." Biological Psychiatry: Cognitive Neuroscience and Neuroimaging.

Cisler, J., B. Sigel, J. Steele, S. Smitherman, K. Vanderzee, J. Pemberton, T. Kramer and C. Kilts (2016). "Changes in functional connectivity of the amygdala during cognitive reappraisal predict symptom reduction during trauma-focused cognitive–behavioral therapy among adolescent girls with post-traumatic stress disorder." Psychological medicine **46**(14): 3013-3023.

Contreras-Rodríguez, O., N. Albein-Urios, J. M. Martinez-Gonzalez, J. M. Menchón, C. Soriano-Mas and A. Verdejo-García (2020). "The neural interface between negative emotion regulation and motivation for change in cocaine dependent individuals under treatment." Drug and Alcohol Dependence: 107854.

Corbalán, F., S. Beaulieu and J. Armony (2015). "Emotion regulation in bipolar disorder type I: an fMRI study." Psychological medicine **45**(12): 2521-2531.

Davis, E. G., L. C. Foland-Ross and I. H. Gotlib (2018). "Neural correlates of top-down regulation and generation of negative affect in major depressive disorder." Psychiatry Research: Neuroimaging **276**: 1-8.

De Wit, S., Y. Van der Werf, D. Mataix-Cols, J. P. Trujillo, P. Van Oppen, D. Veltman and O. Van Den Heuvel (2015). "Emotion regulation before and after transcranial magnetic stimulation in obsessive compulsive disorder." Psychological Medicine **45**(14): 3059-3073.

Denny, B. T., J. Fan, X. Liu, K. N. Ochsner, S. Guerreri, S. J. Mayson, L. Rimsky, A. McMaster, A. S. New and M. Goodman (2015). "Elevated amygdala activity during reappraisal anticipation predicts anxiety in avoidant personality disorder." Journal of affective disorders **172**: 1-7.

Dillon, D. G. and D. A. Pizzagalli (2013). "Evidence of successful modulation of brain activation and subjective experience during reappraisal of negative emotion in unmedicated depression." Psychiatry Research: Neuroimaging **212**(2): 99-107.

Dixon, M. L., C. A. Moodie, P. R. Goldin, N. Farb, R. G. Heimberg and J. J. Gross (2020). "Emotion Regulation in Social Anxiety Disorder: Reappraisal and Acceptance of Negative Self-beliefs." Biological Psychiatry: Cognitive Neuroscience and Neuroimaging **5**(1): 119-129.

Doré, B. P., O. Rodrik, C. Boccagno, A. Hubbard, J. Weber, B. Stanley, M. A. Oquendo, J. M. Miller, M. E. Sublette and J. J. Mann (2018). "Negative autobiographical memory in depression reflects elevated amygdala-hippocampal reactivity and hippocampally associated emotion regulation." Biological Psychiatry: Cognitive Neuroscience and Neuroimaging **3**(4): 358-366.

Douw, L., M. Quaak, S. M. Fitzsimmons, S. J. de Wit, Y. D. van der Werf, O. A. van den Heuvel and C. Vriend (2020). "Static and dynamic network properties of the repetitive transcranial magnetic stimulation target predict changes in emotion regulation in obsessive-compulsive disorder." Brain stimulation **13**(2): 318-326.

Erk, S., A. Mikschl, S. Stier, A. Ciaramidaro, V. Gapp, B. Weber and H. Walter (2010). "Acute and sustained effects of cognitive emotion regulation in major depression." Journal of Neuroscience **30**(47): 15726-15734.

Felder, J. N., M. J. Smoski, R. V. Kozink, B. Froeliger, J. McClernon, J. Bizzell, C. Petty and G. S. Dichter (2012). "Neural mechanisms of subclinical depressive symptoms in women: a pilot functional brain imaging study." BMC psychiatry **12**(1): 152.

Fitzgerald, J. M., H. Klumpp, S. Langenecker and K. L. Phan (2019). "Transdiagnostic neural correlates of volitional emotion regulation in anxiety and depression." Depression and anxiety **36**(5): 453-464.

Fitzgerald, J. M., A. MacNamara, A. E. Kennedy, C. A. Rabinak, S. A. Rauch, I. Liberzon and K. L. Phan (2017). "Individual differences in cognitive reappraisal use and emotion regulatory brain function in combat‐exposed veterans with and without PTSD." Depression and anxiety **34**(1): 79-88.

Fitzgerald, J. M., K. L. Phan, A. E. Kennedy, S. A. Shankman, S. A. Langenecker and H. Klumpp (2017). "Prefrontal and amygdala engagement during emotional reactivity and regulation in generalized anxiety disorder." Journal of affective disorders **218**: 398-406.

Fonzo, G. A., M. S. Goodkind, D. J. Oathes, Y. V. Zaiko, M. Harvey, K. K. Peng, M. E. Weiss, A. L. Thompson, S. E. Zack and S. E. Lindley (2017). "PTSD psychotherapy outcome predicted by brain activation during emotional reactivity and regulation." American Journal of Psychiatry **174**(12): 1163-1174.

Fonzo, G. A., M. S. Goodkind, D. J. Oathes, Y. V. Zaiko, M. Harvey, K. K. Peng, M. E. Weiss, A. L. Thompson, S. E. Zack and C. E. Mills-Finnerty (2017). "Selective effects of psychotherapy on frontopolar cortical function in PTSD." American Journal of Psychiatry **174**(12): 1175-1184.

Gaebler, M., J. K. Daniels, J.-P. Lamke, T. Fydrich and H. Walter (2014). "Behavioural and neural correlates of self-focused emotion regulation in social anxiety disorder." Journal of psychiatry & neuroscience: JPN **39**(4): 249.

Goldin, P., M. Ziv, H. Jazaieri, K. Hahn and J. J. Gross (2013). "MBSR vs aerobic exercise in social anxiety: fMRI of emotion regulation of negative self-beliefs." Social cognitive and affective neuroscience **8**(1): 65-72.

Goldin, P. R., M. Ziv, H. Jazaieri, K. Hahn, R. Heimberg and J. J. Gross (2013). "Impact of cognitive behavioral therapy for social anxiety disorder on the neural dynamics of cognitive reappraisal of negative self-beliefs: randomized clinical trial." JAMA psychiatry **70**(10): 1048-1056.

Goldin, P. R., M. Ziv, H. Jazaieri, J. Weeks, R. G. Heimberg and J. J. Gross (2014). "Impact of cognitive-behavioral therapy for social anxiety disorder on the neural bases of emotional reactivity to and regulation of social evaluation." Behaviour research and therapy **62**: 97-106.

Gorka, S. M., C. B. Young, H. Klumpp, A. E. Kennedy, J. Francis, O. Ajilore, S. A. Langenecker, S. A. Shankman, M. G. Craske and M. B. Stein (2019). "Emotion-based brain mechanisms and predictors for SSRI and CBT treatment of anxiety and depression: a randomized trial." Neuropsychopharmacology **44**(9): 1639-1648.

Heissler, J., P. Kanske, S. Schönfelder and M. Wessa (2014). "Inefficiency of emotion regulation as vulnerability marker for bipolar disorder: evidence from healthy individuals with hypomanic personality." Journal of affective disorders **152**: 83-90.

Heller, A. S., T. Johnstone, M. J. Peterson, G. G. Kolden, N. H. Kalin and R. J. Davidson (2013). "Increased prefrontal cortex activity during negative emotion regulation as a predictor of depression symptom severity trajectory over 6 months." JAMA psychiatry **70**(11): 1181-1189.

Hermann, A., V. Leutgeb, W. Scharmüller, D. Vaitl, A. Schienle and R. Stark (2013). "Individual differences in cognitive reappraisal usage modulate the time course of brain activation during symptom provocation in specific phobia." Biology of mood & anxiety disorders **3**(1): 16.

Hermann, A., A. Schäfer, B. Walter, R. Stark, D. Vaitl and A. Schienle (2009). "Emotion regulation in spider phobia: role of the medial prefrontal cortex." Social cognitive and affective neuroscience **4**(3): 257-267.

Hilland, E., N. Landrø, C. Harmer, M. Browning, L. A. Maglanoc and R. Jonassen (2018). "Attentional bias modification alters fMRI response towards negative stimuli in residual depression." bioRxiv: 322842.

Jacob, Y., O. Shany, P. Goldin, J. Gross and T. Hendler (2019). "Reappraisal of interpersonal criticism in social anxiety disorder: A brain network hierarchy perspective." Cerebral Cortex **29**(7): 3154-3167.

Jansen, J. M., O. Van Den Heuvel, Y. D. van der Werf, S. J. De Wit, D. J. Veltman, W. Van Den Brink and A. E. Goudriaan (2019). "Emotion processing, reappraisal and craving in alcohol dependence: a functional Magnetic Resonance Imaging study." Frontiers in psychiatry **10**: 227.

Jansen, J. M., O. A. van den Heuvel, Y. D. van der Werf, S. J. de Wit, D. J. Veltman, W. van den Brink and A. E. Goudriaan (2019). "The effect of high-frequency repetitive transcranial magnetic stimulation on emotion processing, reappraisal, and craving in alcohol use disorder patients and healthy controls: a functional magnetic resonance imaging study." Frontiers in psychiatry **10**.

Kanske, P., J. Heissler, S. Schönfelder and M. Wessa (2012). "Neural correlates of emotion regulation deficits in remitted depression: the influence of regulation strategy, habitual regulation use, and emotional valence." Neuroimage **61**(3): 686-693.

Kanske, P., S. Schönfelder, J. Forneck and M. Wessa (2015). "Impaired regulation of emotion: neural correlates of reappraisal and distraction in bipolar disorder and unaffected relatives." Translational psychiatry **5**(1): e497-e497.

Karim, H., D. Tudorascu, M. Butters, S. Walker, H. Aizenstein and C. Andreescu (2017). "In the grip of worry: cerebral blood flow changes during worry induction and reappraisal in late-life generalized anxiety disorder." Translational psychiatry **7**(8): e1204-e1204.

Klumpp, H., J. M. Fitzgerald, K. L. Kinney, A. E. Kennedy, S. A. Shankman, S. A. Langenecker and K. L. Phan (2017). "Predicting cognitive behavioral therapy response in social anxiety disorder with anterior cingulate cortex and amygdala during emotion regulation." NeuroImage: Clinical **15**: 25-34.

Klumpp, H., K. L. Kinney, R. Bhaumik and J. M. Fitzgerald (2019). "Principal component analysis and brain-based predictors of emotion regulation in anxiety and depression." Psychological medicine **49**(14): 2320-2329.

Klumpp, H., J. Roberts, M. C. Kapella, A. E. Kennedy, A. Kumar and K. L. Phan (2017). "Subjective and objective sleep quality modulate emotion regulatory brain function in anxiety and depression." Depression and anxiety **34**(7): 651-660.

Klumpp, H., J. Roberts, A. E. Kennedy, S. A. Shankman, S. A. Langenecker, J. J. Gross and K. L. Phan (2017). "Emotion regulation related neural predictors of cognitive behavioral therapy response in social anxiety disorder." Progress in Neuro-Psychopharmacology and Biological Psychiatry **75**: 106-112.

Kreifelts, B., C. Brück, T. Ethofer, J. Ritter, L. Weigel, M. Erb and D. Wildgruber (2017). "Prefrontal mediation of emotion regulation in social anxiety disorder during laughter perception." Neuropsychologia **96**: 175-183.

Lang, S., B. Kotchoubey, C. Frick, C. Spitzer, H. J. Grabe and S. Barnow (2012). "Cognitive reappraisal in trauma-exposed women with borderline personality disorder." Neuroimage **59**(2): 1727-1734.

LeWinn, K. Z., I. A. Strigo, C. G. Connolly, T. C. Ho, O. Tymofiyeva, M. D. Sacchet, H. Y. Weng, E. H. Blom, A. N. Simmons and T. T. Yang (2018). "An exploratory examination of reappraisal success in depressed adolescents: Preliminary evidence of functional differences in cognitive control brain regions." Journal of affective disorders **240**: 155-164.

Light, S. N., A. S. Heller, T. Johnstone, G. G. Kolden, M. J. Peterson, N. H. Kalin and R. J. Davidson (2011). "Reduced right ventrolateral prefrontal cortex activity while inhibiting positive affect is associated with improvement in hedonic capacity after 8 weeks of antidepressant treatment in major depressive disorder." Biological psychiatry **70**(10): 962-968.

Loeffler, L. A. K., T. D. Satterthwaite, U. Habel, F. Schneider, S. Radke and B. Derntl (2019). "Attention control and its emotion-specific association with cognitive emotion regulation in depression." Brain imaging and behavior **13**(6): 1766-1779.

Materna, L., C. D. Wiesner, A. Shushakova, J. Trieloff, N. Weber, A. Engell, R. I. Schubotz, J. Bauer, A. Pedersen and P. Ohrmann (2019). "Adult patients with ADHD differ from healthy controls in implicit, but not explicit, emotion regulation." Journal of psychiatry & neuroscience: JPN **44**(5): 340.

Meluken, I., N. M. Ottesen, K. L. Phan, P. R. Goldin, M. Di Simplicio, J. Macoveanu, H. R. Siebner, L. V. Kessing, M. Vinberg and K. W. Miskowiak (2019). "Neural response during emotion regulation in monozygotic twins at high familial risk of affective disorders." NeuroImage: Clinical **21**: 101598.

Modinos, G., J. Ormel and A. Aleman (2010). "Altered activation and functional connectivity of neural systems supporting cognitive control of emotion in psychosis proneness." Schizophrenia research **118**(1-3): 88-97.

Murphy, E. R., D. M. Barch, D. Pagliaccio, J. L. Luby and A. C. Belden (2016). "Functional connectivity of the amygdala and subgenual cingulate during cognitive reappraisal of emotions in children with MDD history is associated with rumination." Developmental cognitive neuroscience **18**: 89-100.

Perlman, G., A. N. Simmons, J. Wu, K. S. Hahn, S. F. Tapert, J. E. Max, M. P. Paulus, G. G. Brown, G. K. Frank and L. Campbell-Sills (2012). "Amygdala response and functional connectivity during emotion regulation: a study of 14 depressed adolescents." Journal of affective disorders **139**(1): 75-84.

Pitskel, N. B., D. Z. Bolling, M. D. Kaiser, K. A. Pelphrey and M. J. Crowley (2014). "Neural systems for cognitive reappraisal in children and adolescents with autism spectrum disorder." Developmental cognitive neuroscience **10**: 117-128.

Platt, B., C. A. Campbell, A. C. James, S. E. Murphy, M. J. Cooper and J. Y. Lau (2015). "Cognitive reappraisal of peer rejection in depressed versus non-depressed adolescents: functional connectivity differences." Journal of psychiatric research **61**: 73-80.

Rabinak, C. A., A. MacNamara, A. E. Kennedy, M. Angstadt, M. B. Stein, I. Liberzon and K. L. Phan (2014). "Focal and aberrant prefrontal engagement during emotion regulation in veterans with posttraumatic stress disorder." Depression and anxiety **31**(10): 851-861.

Raschle, N. M., L. V. Fehlbaum, W. M. Menks, A. Martinelli, M. Prätzlich, A. Bernhard, K. Ackermann, C. Freitag, S. De Brito and G. Fairchild (2019). "Atypical Dorsolateral Prefrontal Activity in Female Adolescents With Conduct Disorder During Effortful Emotion Regulation." Biological Psychiatry: Cognitive Neuroscience and Neuroimaging **4**(11): 984-994.

Reinecke, A., K. Thilo, N. Filippini, A. Croft and C. J. Harmer (2014). "Predicting rapid response to cognitive-behavioural treatment for panic disorder: the role of hippocampus, insula, and dorsolateral prefrontal cortex." Behaviour research and therapy **62**: 120-128.

Reinecke, A., K. V. Thilo, A. Croft and C. J. Harmer (2018). "Early effects of exposure-based cognitive behaviour therapy on the neural correlates of anxiety." Translational psychiatry **8**(1): 1-9.

Rive, M. M., R. J. Mocking, M. W. Koeter, G. van Wingen, S. J. de Wit, O. A. van den Heuvel, D. J. Veltman, H. G. Ruhé and A. H. Schene (2015). "State-dependent differences in emotion regulation between unmedicated bipolar disorder and major depressive disorder." JAMA psychiatry **72**(7): 687-696.

Rubin-Falcone, H., J. Weber, R. Kishon, K. Ochsner, L. Delaparte, B. Doré, S. Raman, B. T. Denny, M. A. Oquendo and J. J. Mann (2020). "Neural predictors and effects of cognitive behavioral therapy for depression: the role of emotional reactivity and regulation." Psychological medicine **50**(1): 146-160.

Rubin-Falcone, H., J. Weber, R. Kishon, K. Ochsner, L. Delaparte, B. Doré, F. Zanderigo, M. A. Oquendo, J. J. Mann and J. M. Miller (2018). "Longitudinal effects of cognitive behavioral therapy for depression on the neural correlates of emotion regulation." Psychiatry Research: Neuroimaging **271**: 82-90.

Schmitgen, M. M., I. Niedtfeld, R. Schmitt, F. Mancke, D. Winter, C. Schmahl and S. C. Herpertz (2019). "Individualized treatment response prediction of dialectical behavior therapy for borderline personality disorder using multimodal magnetic resonance imaging." Brain and Behavior **9**(9): e01384.

Schmitt, R., D. Winter, I. Niedtfeld, S. C. Herpertz and C. Schmahl (2016). "Effects of psychotherapy on neuronal correlates of reappraisal in female patients with borderline personality disorder." Biological Psychiatry: Cognitive Neuroscience and Neuroimaging **1**(6): 548-557.

Seidel, M., J. A. King, F. Ritschel, I. Boehm, D. Geisler, F. Bernardoni, M. Beck, S. Pauligk, R. Biemann and A. Strobel (2018). "Processing and regulation of negative emotions in anorexia nervosa: an fMRI study." NeuroImage: Clinical **18**: 1-8.

Seidel, M., J. A. King, F. Ritschel, I. Boehm, D. Geisler, F. Bernardoni, L. Holzapfel, S. Diestel, K. Diers and A. Strobel (2018). "The real-life costs of emotion regulation in anorexia nervosa: a combined ecological momentary assessment and fMRI study." Translational psychiatry **8**(1): 1-11.

Simsek, F., K. Oguz, O. Kitis, S. Akan, M. Kempton and A. Gonul (2017). "Neural activation during cognitive reappraisal in girls at high risk for depression." Progress in Neuro-Psychopharmacology and Biological Psychiatry **77**: 49-56.

Van Der Velde, J., E. M. Opmeer, E. J. Liemburg, R. Bruggeman, R. Nieboer, L. Wunderink and A. Aleman (2015). "Lower prefrontal activation during emotion regulation in subjects at ultrahigh risk for psychosis: an fMRI-study." npj Schizophrenia **1**(1): 1-7.

Walsh, E. C., T. A. Eisenlohr-Moul, J. Minkel, J. Bizzell, C. Petty, A. Crowther, H. Carl, M. J. Smoski and G. S. Dichter (2019). "Pretreatment brain connectivity during positive emotion upregulation predicts decreased anhedonia following behavioral activation therapy for depression." Journal of affective disorders **243**: 188-192.

Wang, X., Z. Feng, D. Zhou, X. Lei, T. Liao, L. Zhang, B. Ji and J. Li (2014). "Dissociable self effects for emotion regulation: a study of chinese major depressive outpatients." BioMed research international **2014**.

Yip, S. W., J. J. Gross, M. Chawla, S.-S. Ma, X.-H. Shi, L. Liu, Y.-W. Yao, L. Zhu, P. D. Worhunsky and J. Zhang (2018). "Is neural processing of negative stimuli altered in addiction independent of drug effects? Findings from drug-naïve youth with internet gaming disorder." Neuropsychopharmacology **43**(6): 1364-1372.

Young, K. S., R. T. LeBeau, A. N. Niles, K. J. Hsu, L. J. Burklund, B. Mesri, D. Saxbe, M. D. Lieberman and M. G. Craske (2019). "Neural connectivity during affect labeling predicts treatment response to psychological therapies for social anxiety disorder." Journal of affective disorders **242**: 105-110.

Zhang, J., H. Dong, Z. Zhao, S. Chen, Q. Jiang, X. Du and G.-H. Dong (2020). "Altered Neural Processing of Negative Stimuli in People with Internet Gaming Disorder: fMRI Evidence from the Comparison with Recreational Game Users." Journal of Affective Disorders.

Zhang, L., E. M. Opmeer, L. van der Meer, A. Aleman, B. Ćurčić‐Blake and H. G. Ruhé (2018). "Altered frontal‐amygdala effective connectivity during effortful emotion regulation in bipolar disorder." Bipolar disorders **20**(4): 349-358.
